# Mobile Apps Addressing Feeding of Children With Autism: Systematic Search and Content and Quality Analysis

**DOI:** 10.2196/94908

**Published:** 2026-11-10

**Authors:** Fabrice Mukarage, Christopher Cofie Kuzagbe, Skye Nandi Adams, N’guessan Yves-Roland Douha, Edith Talina Luhanga

**Affiliations:** 1 Carnegie Mellon University Africa, Special Economic Zone, Regional ICT Center of Excellence, Kigali, Rwanda, +250 791 590 206; 2 University of the Witwatersrand, Johannesburg, Gauteng, South Africa

**Keywords:** autism spectrum disorder, ASD, mobile health, mHealth, caregiver support, behavior change wheel, Mobile App Rating Scale, MARS

## Abstract

**Background:**

Approximately 1 in 100 children worldwide are diagnosed with autism spectrum disorder (ASD), and 46% to 89% of those diagnosed experience significant feeding difficulties. Mobile health (mHealth) apps are a potential tool for scalable support. However, there is nascent literature on the quality and relevance of mHealth apps for managing ASD-related feeding challenges.

**Objective:**

This study aimed at systematically identifying and reviewing the content, behavior change strategies, and quality of free English-language mHealth apps supporting the feeding of children with autism.

**Methods:**

A systematic search was conducted on the Apple App Store and Google Play Store between September and October 2024 and updated between June and July 2026. Apps were included if they were free or freemium, in English, updated within the past year, and explicitly focused on the feeding of children with autism. Apps were excluded if they targeted clinicians rather than parents or children; could not be installed on the test devices; or primarily focused on learning or communication rather than feeding, despite descriptions suggesting relevance to feeding. We identified intervention strategies in the included apps using the behavior change wheel (BCW) framework and evaluated their quality using the Mobile App Rating Scale (MARS).

**Results:**

The search returned 333 apps from both app stores. After removing duplicates and screening, only 2 iOS apps (EduKitchen-Toddlers Food Games and Autism Food Coach 2) met all inclusion criteria. 331 apps were excluded due to their focus on communication, early education, or other autism support that was not food related. EduKitchen-Toddlers Food Games provided child-centered interactive games and sensory-friendly visuals, while Autism Food Coach 2 provided structured caregiver tools, visual meal plans, and progress tracking. EduKitchen-Toddlers Food Games provided the BCW intervention functions of education, training, enablement, incentivization, and modeling, while Autism Food Coach 2 provided education, training, enablement, persuasion, incentivization, and environmental restructuring. The apps received MARS scores of 3.9 and 3.7, respectively, indicating acceptable quality. The apps’ shortcomings included limited customization for diverse user needs and the absence of documented clinical validation.

**Conclusions:**

There is a critical shortage of mHealth apps for feeding difficulties in children with autism that are both evidence-based and of high quality. Future development must integrate robust clinical validation and comprehensive, caregiver-centered support features to address this significant gap.

## Introduction

Autism spectrum disorder (ASD) is a complex neurodevelopmental condition characterized by challenges in social interaction, communication, behavior regulation, and sensory processing. The global prevalence of ASD has increased significantly in recent decades; the US Centers for Disease Control and Prevention reports a 178% increase in autism prevalence since 2000 [1], and the World Health Organization estimates that approximately 1 in 100 children is diagnosed with ASD [2].

Among the various comorbidities observed in children with autism, feeding difficulties are especially common and underaddressed. Studies report prevalence rates ranging from 46% to 89% [3], with affected children exhibiting food selectivity, aversions to textures and flavors, rapid eating, and disruptive mealtime behaviors [4,5]. These challenges are linked to nutritional deficiencies, impaired growth, gastrointestinal disorders, and metabolic imbalances [6,7]. Children with autism experience feeding problems 5 times more frequently than their neurotypical peers [8]. This situation places a significant burden on caregivers and parents, who often serve as the primary facilitators of feeding interventions. They manage complex feeding routines while navigating children’s behavioral and communication challenges. Given their central role in daily feeding routines and their intimate knowledge of their child’s behaviors, parents represent a critical intervention point for addressing these challenges.

Parent-mediated interventions are a prominent model for supporting children with autism. Previous research indicates that parents can successfully learn and implement intervention strategies [9]. However, translating these interventions into community settings remains challenging due to persistent barriers in accessibility and scalability [10]. The emergence of mobile health (mHealth) apps presents a promising means to overcome these barriers. A growing body of research shows mHealth apps can effectively train parents and improve child outcomes in areas such as communication, socialization, and behavior management [11,12].

The success of these digital tools, however, is contingent upon their design and usability. Research indicates that parent engagement is directly linked to an app’s perceived utility and its alignment with daily caregiving realities [13]. Poorly designed interventions that fail to account for parental needs, stress levels, and caregiving contexts may inadvertently exacerbate caregiver burden [14,15]. Indeed, systematic reviews of mHealth apps across domains such as child nutrition, child growth monitoring, and infant care show considerable variations in app quality [16,17]. Most apps have acceptable to good quality when evaluated with standardized tools such as the Mobile App Rating Scale (MARS) [18], largely due to lower engagement, usability, or quality of information scores. Other reviews have identified limited incorporation of evidence-based health information and behavior change practices in child health and parenting apps [16,17]. These findings highlight the necessity of systematically evaluating digital health interventions rather than assuming their effectiveness based on availability alone.

However, despite the growing interest in digital interventions for autism and the variable reported quality of mHealth apps, research has scarcely explored the range, support features, and quality of mHealth apps specifically designed to help parents manage feeding difficulties. The aim of this study was to conduct a systematic search and evaluation of the content, behavior change strategies, and quality of free English-language apps supporting the feeding of children with autism in the 2 major app stores (Google Play Store and Apple App Store). For included apps, we identified informational topics and app features and mapped them to the behavior change wheel (BCW) [19] to determine the scope and the supported behavior change intervention functions. The BCW provides a comprehensive theoretical framework for analyzing behavior change potential through intervention functions such as education, persuasion, and incentivization. Understanding the behavior change strategies embedded in an app is critical to evaluating its likely effectiveness. We then assessed the app quality using the MARS [18], a validated and widely used tool for evaluating mHealth app quality across 4 critical factors: engagement, functionality, aesthetics, and information quality.

Overall, this study answered the following research questions:

1. What is the availability of apps addressing feeding difficulties in children with autism in the 2 major commercial app stores?
2. What behavior change intervention functions are included in available apps?
3. What is the overall quality of available apps when assessed using the MARS?
4. What are the strengths and limitations of existing apps in supporting feeding-related interventions?
5. What actionable recommendations can guide the development of future mHealth apps that more effectively assist caregivers in managing ASD-related feeding difficulties?

## Methods

### Eligibility Criteria

#### Inclusion Criteria

We analyzed apps that met the following inclusion criteria: (1) the app is free to download (either fully free or freemium, ie, offering in-app purchases), (2) the content is written in English, (3) the app has been updated within the past year (to exclude defunct or outdated apps), and (4) the title or description explicitly addresses feeding in children with autism.

We limited inclusion to English-only apps, as it is the most widely spoken language worldwide when combining native and non-native speakers [20]. As researchers based in Africa, this choice also reflected local realities, as English is commonly used as an official or working language in many countries, with 26 countries in sub-Saharan Africa alone using English as an official language, either exclusively or alongside local languages [21].

#### Exclusion Criteria

Apps were excluded if they (1) targeted clinicians rather than caregivers or children; (2) primarily focused on learning or communication rather than feeding, despite descriptions suggesting relevance to feeding; or (3) could not be installed on the test devices (iPhone 12 Pro Max for iOS and Google Pixel 3a XL for Android) due to geographical restrictions, unsupported operating system versions, or other installation challenges.

### Information Sources

Searches were conducted on the Google Play Store and Apple App Store. The Google Play Store holds more than 70% of smartphone apps market share [22], while the Apple App Store is considered the second largest app market store [23].

### Search Strategy

We conducted a systematic search of the Apple App Store (iOS) and Google Play Store (Android) to identify mHealth apps related to feeding, food, meal planning, or nutrition for children with autism. The search was performed between September and October 2024 and updated between June and July 2026. All researchers were based in Rwanda, East Africa, during the study. The search terms used included: “child autism food,” “kids autism food,” “toddler autism food,” “child autism nutrition,” “kids autism nutrition,” “toddler autism nutrition,” “child autism meal prep planning,” “kids autism meal prep planning,” “toddler autism meal prep planning,” “child autism feeding,” “kids autism feeding,” “toddler autism feeding,” “child autism mealtime,” “kids autism mealtime,” and “toddler autism mealtime.” These terms were entered directly into the Apple App Store and Google Play Store search interfaces with no filters or limits applied. The searches were conducted using an iPhone 12 Pro Max (iOS) and a Google Pixel 3a XL (Android) to simulate a typical end-user experience on each platform. All returned results were saved in a Microsoft Excel file for deduplication and screening.

### Selection Process

After removing duplicates, 2 members of the research team independently screened the apps. We undertook a 2-phase screening process. In the first phase, the apps’ information pages (screenshots and textual description) were screened against the inclusion and exclusion criteria. Apps with a clearly unrelated scope (eg, communication support only) were excluded. Apps that included child feeding as 1 of several features or suggested a broader scope of features than those listed on the page were moved to phase 2. In this phase, the apps were downloaded and installed on the test devices. They were then screened to determine if they contained feeding-related content. Apps that had feeding-related content were included in the final analysis. The screening results were compared, and disagreements were resolved by discussion or by consulting with a third member of the research team.

### Data Collection Process and Data Items

Data was independently extracted by the same 2 members of the research team who conducted screening, with 1 reviewer focusing on the Apple App Store and the other on the Google Play Store. Reviewers independently navigated all freely accessible pages and recorded the informational content and features in a Microsoft Excel file and then validated each other’s lists. The following data items were recorded per page: informational content (using article headings), interactive features, and whether each feature was free to access or paywalled. To ensure reliability, the reviewers cross-validated each other’s assessments by switching platforms after the initial evaluations.

Deductive coding was then conducted using the BCW [19], with each feature mapped to 1 of the 9 intervention functions: education, persuasion, incentivization, coercion, training, restriction, environmental restructuring, modeling, and enablement. In a preparatory session, both reviewers reviewed the BCW framework and discussed descriptions of the intervention functions to ensure a shared understanding of the categories. They also created examples of app features to ensure consistent coding. Discrepancies were discussed until consensus was reached; when consensus was not possible, a third reviewer was consulted. A deductive approach was selected to assess the extent to which app content aligned with established behavior change theory, rather than to generate novel thematic categories.

### App Quality Assessment

The quality of the apps was assessed using the MARS [18], a validated tool for the evaluation of mHealth apps. MARS evaluates apps on 4 dimensions: engagement (user interest and interaction), functionality (performance and usability), aesthetics (visual design), and quality of information (accuracy, relevance, and credibility of content). Each dimension is rated on a 5-point Likert scale. Two reviewers independently rated each app. Both reviewers have a background in mobile app design and development and had worked on mHealth projects over the past year. The reviewers also completed the official MARS training to ensure consistent interpretation of the scale. Following the independent rating phase, the 2 reviewers discussed their evaluations to reach a consensus score for each MARS item. These final consensus scores were used in the analysis.

### Ethical Considerations

The study did not require any ethics review.

## Results

### App Search and Selection

The initial search (conducted in September and October 2024) yielded 326 apps (27 iOS apps from the Apple App Store and 299 Android apps from the Google Play Store). From these 326 apps, 34 duplicates were removed, leaving 292 apps for phase 1 screening. After this stage, 61 apps were retained for phase 2 screening. The remaining apps were excluded because their focus was preschool education (eg, learning the alphabet, numbers, and animals), communication skills support, or emotional regulation through games or musical therapy. During phase 2 screening, a further 59 apps were excluded, as they had a similar focus on learning and communication. Overall, only 2 iOS apps (EduKitchen-Toddlers Food Games and Autism Food Coach 2) were found to address feeding challenges in children with autism, indicating a critical shortage on the number of available apps for supporting caregivers in the home management of feeding difficulties in children with autism.

Our updated search from June to July 2026 returned 333 apps, with 46 duplicates removed. After applying the inclusion and exclusion criteria, no additional apps were included in the study, showing no improvement in the availability of feeding support apps. We consider the implications of this in the Discussion section. A complete list of the screened apps, including their platform and primary focus, is provided in Table S1 of Multimedia Appendix 1. Figure 1 presents the app selection process following PRISMA (Preferred Reporting Items for Systematic Reviews and Meta-Analyses) guidelines [24].

**Figure 1.**
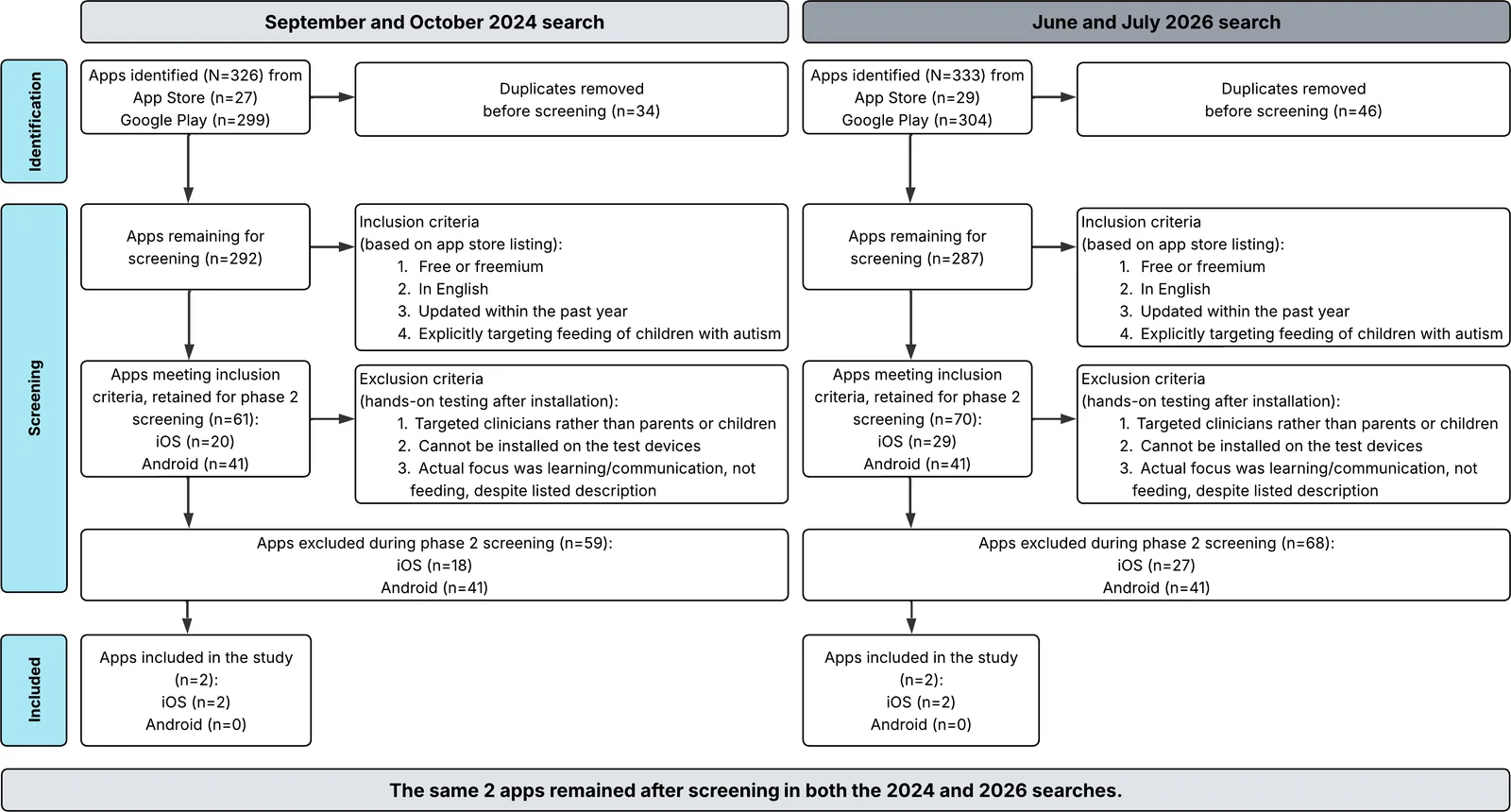
PRISMA (Preferred Reporting Items for Systematic Reviews and Meta-Analyses) flow diagram of mobile health (mHealth) app selection for the systematic review.

### Description of the Selected Apps

Both EduKitchen-Toddlers Food Games and Autism Food Coach 2 are commercial apps, with the former developed by Cubic Frog Apps and the latter by Aidan’s Apps. EduKitchen-Toddlers Food Games is a child-facing app. Its stated aim is to provide a set of kitchen games that encourage wiser food knowledge while also enhancing the cognitive skills emphasized in preschool curricula, such as sorting and matching. Meanwhile, Autism Food Coach 2 is a caregiver- and child-facing app that was developed by a parent to promote slower chewing behavior in their child. Neither of the apps reports any scientific studies conducted during app development or any evaluation studies conducted on efficacy. Similarly, there was no report on the evidence used to inform content and feature selection.

### Evaluation of App Features and Content

EduKitchen-Toddlers Food Games targets early childhood learners and supports preschool cognitive skills of counting, sorting, sequencing, and related cognitive skills alongside building food familiarity through interactive play. The app (Table 1) includes 18 free mini-games focused on cooking and kitchen tasks, for example, frying and counting eggs (Figure 2A) and sorting food-related waste (Figure 2B). The foods covered in the app included whole foods such as eggs, fruits (eg, apples, bananas, and strawberries), vegetables, cereals, bread, and fruit juices, along with processed foods such as cookies, ice cream, popsicles, and sugar-sweetened soft drinks. Although there were no lessons around distinguishing healthy from unhealthy foods, we did note that healthy foods were more prominently represented.

**Table 1.** Core features and access models of the selected apps.

App name	Access model	Target audience	Core features
EduKitchen-Toddlers Food Games	One-time purchase	Toddlers/preschool children including children with autism Parents/caregivers who configure the app (secondary audience)	Gamified food-related activities (eg, sorting, matching, and counting) Reinforcement through animations and in-app rewards Customizable settings for sensory preferences and learning levels Engaging, child-friendly interface with cartoon visuals
Autism Food Coach 2	Subscription (£49.99/month)^a^	Parents and caregivers of children with autism who have feeding difficulties children with autism who participate in feeding routines (secondary audience)	Structured mealtime routines with visual schedules Meal tracking and progress dashboards for caregivers Customizable feeding plans for behavioral and sensory needs Motivational prompts, goal setting, and reward systems

a £1=US $1.32 as of October 5, 2026.

**Figure 2.**
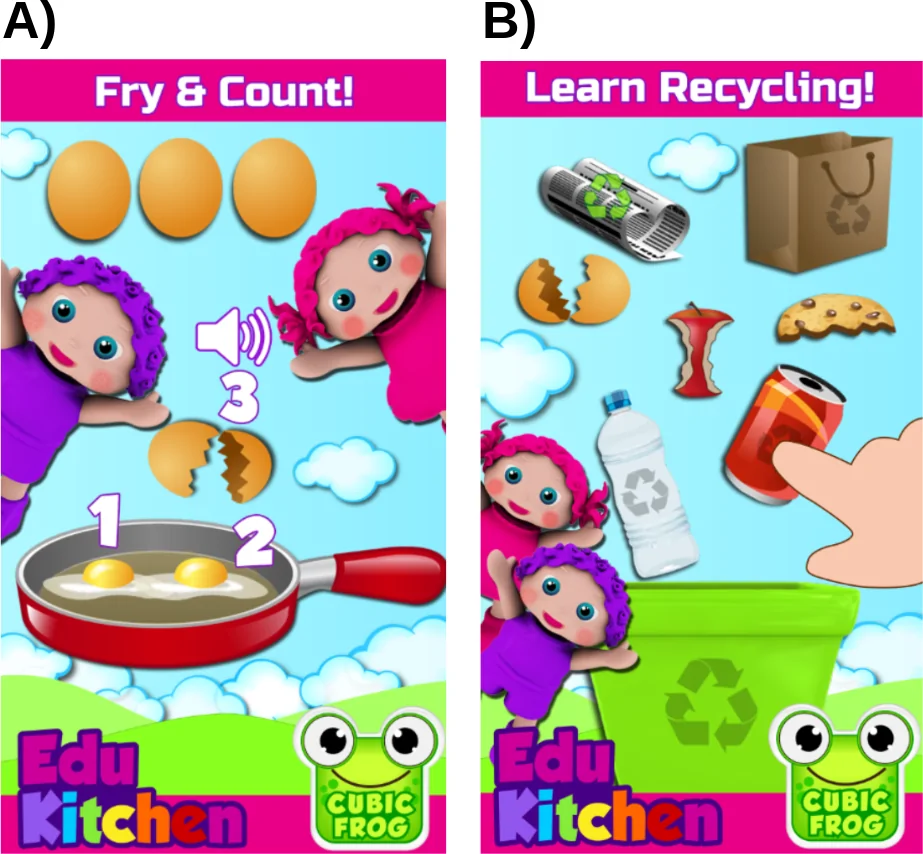
Screenshots from EduKitchen-Toddlers Food Games, developed by Cubic Frog Apps, obtained via the Apple App Store. The app features food-themed games intended to build early familiarity with food through sorting, counting, and categorization. Example games include (A) frying and counting eggs and (B) sorting food-related waste.

Children can interact with the app through touch-based activities guided by voice instructions, with multiple languages supported, while parents can customize the app to select the language, adjust the difficulty level of activities, and unlock the full set of educational games through the premium version. Since the app targets young children, there was no informational content provided.

Autism Food Coach 2 is designed for caregivers and children with autism, providing a structured approach to support feeding routines. As shown in Figure 3, the app includes motivational messages that encourage emotional reflection and sensory modulation during meals. For example, Figure 3A advises users to “notice the effects food has on your feelings,” while Figure 3B suggests playing relaxing music during meals to enhance enjoyment. The app includes additional functionalities, such as progress tracking, visual schedules, and personalized meal planning tools, that allow systematic tracking of mealtime behaviors. Motivational prompts and sensory regulation suggestions (eg, relaxing music or emotional reflection) are also provided to create calmer mealtime environments.

Although both apps incorporate food-related content, they diverge considerably in functional purpose and clinical utility: one offers indirect exposure to foods and feeding support through child-led play, while the other provides direct support for caregiver-led interventions. This section evaluates the extent to which the apps support structured feeding interventions, promote adaptive mealtime behaviors, and guide caregiver decision-making.

We formalize this analytical comparison in Textbox 1, which categorizes app features based on their ability to provide actionable guidance to support caregivers during feeding interventions. The analysis confirms that only Autism Food Coach 2 includes features that align with caregiver-mediated feeding interventions.

**Figure 3.**
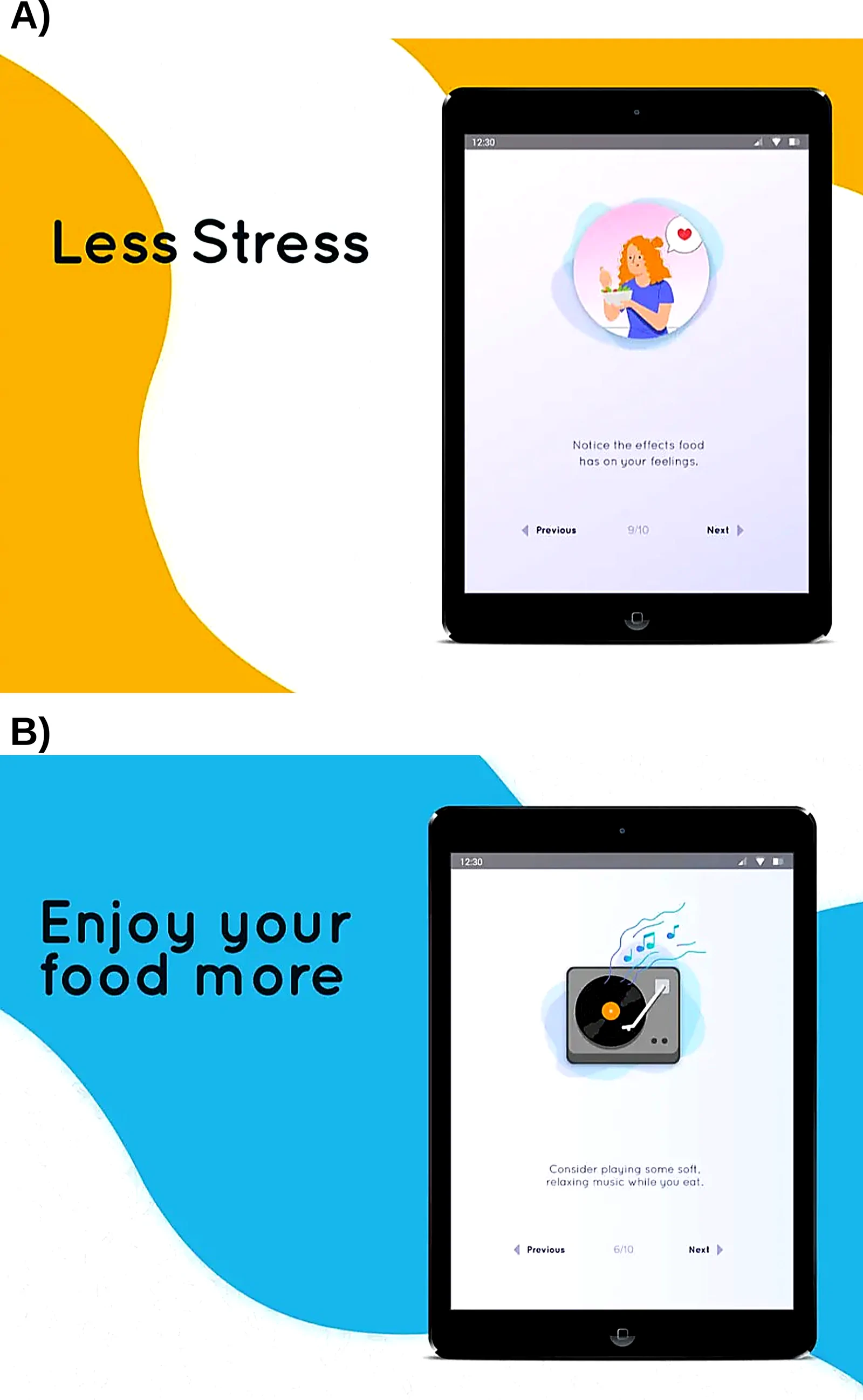
Screenshots from Autism Food Coach 2, developed by Aidan’s Apps, obtained via the Apple App Store. The app includes prompts that, for example, (A) encourage users to reflect on how food affects their feelings and (B) promote calm sensory environments by suggesting soft background music during meals.

Textbox 1. **App features categorized by caregiver decision support capabilities.**

Features with caregiver decision support

- *Instant feedback and rewards* reinforce positive child behaviors, allowing parents to adjust strategies in real time.
- *Parent support tools* offer structured feedback, coaching prompts, and meal tracking to help manage feeding routines.
- *Customizable progress monitoring* provides visual progress charts and behavior tracking to support informed parental decisions.

Features without caregiver decision support

- *Interactive child engagement* focuses on engaging the child through games and narratives but lacks direct caregiver guidance.

### Intervention Function Mapping Using the BCW Framework

The BCW provides a systematic model for designing interventions based on behavioral determinants. Table 2 summarizes the BCW intervention functions identified in each app. We identified 7 of the 9 BCW functions across the 2 apps. It is worth noting that neither app incorporated coercion nor restriction.

**Table 2.** Behavior change wheel intervention function mapping for the 2 reviewed apps.

Intervention function	EduKitchen-Toddlers Food Games	Autism Food Coach 2
Education	✓	✓
Training	✓	✓
Enablement	✓	✓
Persuasion	✗	✓
Incentivization	✓	✓
Modeling	✓	✗
Environmental restructuring	✗	✓
Coercion	✗	✗
Restriction	✗	✗

EduKitchen-Toddlers Food Games mapped onto 5 BCW functions: education, training, enablement, incentivization, and modeling overall, but food-related content mapped onto only 3 interventions. These were education, delivered through interactive lessons introducing food types; incentivization, seen through gamified rewards (eg, praise animations); and modeling, in the use of animated characters demonstrating appropriate feeding behaviors. Features mapped to training and enablement primarily supported preschool learning outcomes. For instance, training is embedded in mini-games that reinforce skills such as sorting and categorization, and enablement is supported via customizable gameplay settings to accommodate diverse sensory or developmental needs.

Autism Food Coach 2 incorporated 6 intervention functions: education, training, enablement, persuasion, incentivization, and environmental restructuring. The app provides educational content through guidance on balanced nutrition and mindful eating. Training is facilitated through structured visual schedules that model consistent feeding routines. Enablement includes personalized meal planning and caregiver-facing tracking tools. Persuasion is operationalized through motivational messages and positive affirmations. Incentivization is achieved through visual progress tracking and behavioral goal setting. Environmental restructuring is implemented through tools that help caregivers create predictable low-stress mealtime environments (eg, use of music, calming messages, and visual schedules).

### MARS Quality Assessment

We present the results of the MARS evaluation for the 2 selected apps. EduKitchen-Toddlers Food Games achieved an overall MARS score of 3.9, reflecting good usability and design quality. It performed strongest in functionality (4.0) due to its stable performance, intuitive navigation, and simple touch-based interactions that allowed young children to complete activities with minimal assistance. A similarly high score was achieved for aesthetics (4.0) because of its colorful, child-friendly interface, large interactive objects, high-contrast visuals, and consistent use of animated characters that maintained children’s attention while reducing visual complexity. The app also incorporated clear audio instructions, simple layouts, and uncluttered screens, making interactions appropriate for preschool-aged children and potentially supportive for children with autism who may benefit from predictable visual environments and multimodal cues. Engagement (3.7) and information quality (3.9) also received strong ratings because the app used a variety of age-appropriate gamified activities, including cooking, counting, sorting, and matching games, to reinforce learning through repeated interaction rather than text-based instruction. However, the information score was slightly lower because educational content was embedded within gameplay and there were no caregiver resources, nutritional guidance, or evidence-based informational materials. The app received a subjective quality score of 4.0, reflecting its suitability as an engaging educational app for young children.

Autism Food Coach 2 achieved an overall MARS score of 3.7, interpreted as acceptable to good usability and design quality. It received its highest ratings for information (3.95) and engagement (3.75). The information score reflected the inclusion of educational modules on mindful eating, digestion, eating pace, emotional awareness during meals, and strategies for improving mealtime experiences, together with interactive features such as personalized eating timers, bite pacing, and meal-type selection. These features encouraged active user participation and supported behavior change throughout the eating process. Functionality (3.8) was rated highly because of the app’s intuitive navigation, responsive performance, and customizable meal timer that could be adapted to different food types or user preferences. Aesthetics (3.3) received the lowest score due to its relatively simple and utilitarian interface, which prioritized functionality over visual engagement. Although the app included clear graphics and a consistent layout, it offered limited animation, interactive visual elements, and child-oriented design features. The app received a subjective quality score of 4.0, reflecting its perceived usefulness as a behavioral support tool for improving eating pace and mindful eating habits.

Table 3 summarizes the scores for the apps across the 4 MARS quality dimensions, that is, engagement, functionality, aesthetics, and information, along with the overall mean and subjective quality ratings. The table highlights a significant difference in the aesthetics dimension, in which EduKitchen-Toddlers Food Games received a substantially higher score (4.0 compared to 3.3). In contrast, both apps performed comparably in engagement, functionality, and information, with differences of 0.2 points or less.

**Table 3.** Mobile App Rating Scale results for the 2 reviewed apps. Each dimension was scored on a 5-point scale.

Characteristic and dimension	EduKitchen-Toddlers Food Games	Autism Food Coach 2
Platform	iOS	iOS
Overall score (mean)	3.9	3.7
Engagement	3.7	3.75
Functionality	4.0	3.8
Aesthetics	4.0	3.3
Information	3.9	3.95
Subjective quality score	4.0	4.0

## Discussion

### Principal Findings

This review examined the Apple App Store and Google Play Store for mobile apps that address feeding challenges in children with autism. Our most significant finding is the scarcity of feeding support apps. Only 2 of more than 300 identified apps met our inclusion criteria, and the same 2 qualified in both search rounds (2024 and 2026). Most excluded apps focused on communication, early education, or other autism support that was not food related. Delayed speech, language impairments, and difficulty with reading and writing are common in autism. Around 30% of children with autism remain nonverbal [25], and many perform worse academically than their IQ would predict [26]. These challenges are serious [27], but feeding difficulties also affect a large share of children with autism, with prevalence estimates of 46% to 89% [3]. Poor nutrition affects physical growth and is linked to long-term cognitive impairment [28]. The emphasis on communication and learning among the autism apps we screened does not match the daily reality of raising a child with autism. Feeding is a daily need for many families, but almost no evidence-based digital support exists for it; mHealth should give feeding the same priority as communication and learning.

The 2 included apps differ in target users and intervention logic. Neither provided a theoretical basis for its features, and we found no documentation of efficacy. Both mapped to 4 core BCW functions: education, training, enablement, and incentivization. For EduKitchen-Toddlers Food Games, training and enablement served preschool learning rather than feeding. These functions match needs described in prior work. Education and training target the capability barriers of teachers and caregivers, who report limited knowledge of feeding strategies for children with autism [29-31]. Enablement helps reduce structural barriers to professional care, which are common in lower-resource settings [32]. Even so, broad BCW coverage did not produce broad support. EduKitchen-Toddlers Food Games used child-centered gamification to build food familiarity, and Autism Food Coach 2 offered basic caregiver tools for meal planning and mindful eating. Established programs such as BUFFET (Building Up Food Flexibility and Exposure Treatment) [33] and the Autism MEAL (Managing Eating Aversions and Limited Variety) Plan [34] combine family-based therapy, gradual food exposure, and sensory integration [35]. The apps offered far less. Limited evidence-based content is also common in nutrition and infant feeding apps more broadly [16].

Both apps also had acceptable but not high-quality scores on the MARS. EduKitchen-Toddlers Food Games scored 3.9 and Autism Food Coach 2 scored 3.7. Earlier MARS evaluations of child nutrition and feeding apps reported overall scores of about 3.0 to 4.0 out of 5 [16,17,36], so our evaluated apps fall in the upper part of that range. Those studies also reported limited engagement features, weak evidence-based content, poor aesthetic design, and little personalization. We found similar limitations (Table 4). Neither app reported clinical validation. EduKitchen-Toddlers Food Games offered basic customization, and Autism Food Coach 2 had the lowest aesthetics score (3.3), reflecting a simple, utilitarian interface.

**Table 4.** Strengths and limitations of the 2 reviewed apps.

	EduKitchen-Toddlers Food Games	Autism Food Coach 2
Strengths	Provides an engaging and interactive learning experience with high-quality graphics and food-themed mini-games; includes basic caregiver customization	Offers tailored meal plans, progress tracking, and structured coaching tools that support caregiver-led feeding interventions
Limitations	No scientific validation; limited features for caregiver-led interventions or clinical feeding challenges	Limited visual design quality; lacks empirical validation through clinical trials

Closing this gap starts with bringing established feeding programs such as BUFFET and the Autism MEAL Plan into digital tools. Building an effective mHealth app requires several stakeholders. Clinicians, such as dietitians and occupational and speech therapists, can keep the clinical content faithful to the original programs. Caregivers know what fits into daily mealtimes, and poorly designed tools can add to their burden [14,15]. Developers can turn this input into features that families find easy and engaging to use. Researchers should then evaluate the tools.

We make 3 recommendations to support this work. First, developers should use structured co-design frameworks (eg, IDEAS [integrate, design, assess, and share] [37]) so that clinical experts and caregivers shape the tools from the start. This helps apps combine evidence-based strategies with informative, engaging features that serve caregivers and children. Second, quick reference guides and practical tutorials on behavioral theory would help developers and stakeholders during rapid ideation sessions, because comprehensive frameworks and training manuals can be hard to navigate. Third, researchers and developers should document feature efficacy through mixed methods evaluations. Quantitative measures, such as food variety and mealtime behavior, show whether a tool improves feeding outcomes. Qualitative methods show how caregivers and children use each feature and why some work better than others. Both are needed because clinical strategies do not always work the same way when delivered digitally [38].

Evidence to guide app design also remains limited, particularly across cultural contexts. Preferences for gamification vary widely with cultural values and social norms [39,40], but little is known about which gamification and engagement features work best in mHealth apps in different settings. Evidence is also thin on how to balance personalization with privacy for different users, such as privacy-conscious users and open sharers. Future research should address these questions. This work is most urgent in low-resource settings, where in-person feeding services are scarce [32] and digital tools could reach families who cannot access them [10-12]. This review provides a baseline against which to track how the app market changes.

### Limitations

While this review provides a structured assessment of the current landscape, several limitations must be considered when interpreting these findings. App search results vary depending on the geographic location. As the research was conducted in Rwanda, the apps available are likely different from those accessible from other countries and regions. This search also used English keywords and only included apps available in English. To fully understand the scope of apps available to the African population, the search should be expanded to include French, Portuguese, Arabic, Spanish, and local languages. Although extensive search terms were used, some relevant apps may have been missed due to alternative labeling by developers. Furthermore, restricted features behind paywalls could not be fully evaluated. Lastly, while MARS provides a standardized assessment from a rater perspective, it may not fully capture caregiver preferences and usability. Future work could consider user-centered measures, such as the user version of the Mobile App Rating Scale (uMARS) [41], to assess app acceptability and perceived usefulness.

Future research should broaden the search strategy to mitigate geographic sampling bias, for example, by using multiregion app store accounts to more comprehensively capture global app availability. Co-design approaches that involve parents, clinicians, and speech or occupational therapists will be crucial in ensuring that future apps address both clinical needs and contextual realities. Particularly in resource-constrained settings, culturally tailored and clinically validated mHealth apps are urgently needed to support caregivers to manage ASD-related feeding challenges. Longitudinal studies evaluating behavioral outcomes and caregiver burden can further establish the real-world impact of these digital interventions.

### Conclusions

This study highlights a critical shortage of mHealth apps specifically designed to support feeding challenges in children with autism. Using the BCW and MARS frameworks, we evaluated the functional strengths and theoretical underpinnings of the 2 eligible apps. Although both apps incorporated behavior change techniques and demonstrated moderate to high usability, neither was grounded in clinically validated feeding interventions. These findings underscore the need for user-centered, evidence-based app development that integrates behavioral, sensory, and skill-building supports tailored to the diverse needs of children with autism. Expanding access to effective, clinically validated, and culturally responsive mHealth tools is essential for advancing digital health equity, particularly in low-resource settings where traditional intervention services may be limited.

## Supplementary material

10.2196/94908 Multimedia Appendix 1 List of screened apps.

## Abbreviations

ASD: autism spectrum disorder
BCW: behavior change wheel
BUFFET: Building Up Food Flexibility and Exposure Treatment
IDEAS: integrate, design, assess, and share
MARS: Mobile App Rating Scale
MEAL: Managing Eating Aversions and Limited Variety
mHealth: mobile health
PRISMA: Preferred Reporting Items for Systematic Reviews and Meta-Analyses
uMARS: user version of the Mobile App Rating Scale

## References

[1] (2025). Data and statistics on autism spectrum disorder. CDC.

[2] (2025). Autism. World Health Organization.

[3] Nadon G, Feldman D, Gisel E, Fitzgerald M (2013). Recent Advances in Autism Spectrum Disorders - Volume 1.

[4] Marshall J, Hill RJ, Ziviani J, Dodrill P (2014). Features of feeding difficulty in children with autism spectrum disorder. Int J Speech Lang Pathol.

[5] Leader G, Tuohy E, Chen JL, Mannion A, Gilroy SP (2020). Feeding problems, gastrointestinal symptoms, challenging behavior and sensory issues in children and adolescents with autism spectrum disorder. J Autism Dev Disord.

[6] Liu X, Liu J, Xiong X (2016). Correlation between nutrition and symptoms: nutritional survey of children with autism spectrum disorder in Chongqing, China. Nutrients.

[7] Marinov D, Chamova R, Pancheva R (2024). Micronutrient deficiencies in children with autism spectrum disorders compared to typically developing children – a scoping review. Res Autism Spectr Disord.

[8] Sharp WG, Berry RC, McCracken C (2013). Feeding problems and nutrient intake in children with autism spectrum disorders: a meta-analysis and comprehensive review of the literature. J Autism Dev Disord.

[9] Beaudoin AJ, Sébire G, Couture M (2014). Parent training interventions for toddlers with autism spectrum disorder. Autism Res Treat.

[10] Rojas-Torres LP, Alonso-Esteban Y, Alcantud-Marín F (2020). Early intervention with parents of children with autism spectrum disorders: a review of programs. Children (Basel).

[11] Lee J, Lim J, Kang S (2024). Mobile app-assisted parent training intervention for behavioral problems in children with autism spectrum disorder: pilot randomized controlled trial. JMIR Hum Factors.

[12] Law GC, Neihart M, Dutt A (2018). The use of behavior modeling training in a mobile app parent training program to improve functional communication of young children with autism spectrum disorder. Autism.

[13] Bonnot O, Adrien V, Venelle V, Bonneau D, Gollier-Briant F, Mouchabac S (2021). Mobile app for parental empowerment for caregivers of children with autism spectrum disorders: prospective open trial. JMIR Ment Health.

[14] Houser L, McCarthy M, Lawer L, Mandell D (2014). A challenging fit: employment, childcare, and therapeutic support in families of children with autism spectrum disorders. J Soc Serv Res.

[15] Bradshaw J, Wolfe K, Hock R, Scopano L (2022). Advances in supporting parents in interventions for autism spectrum disorder. Pediatr Clin North Am.

[16] Cheng H, Tutt A, Llewellyn C (2020). Content and quality of infant feeding smartphone apps: five-year update on a systematic search and evaluation. JMIR mHealth uHealth.

[17] Francis L, Spaulding EM, Bloom I, Patel A, Perrin N (2023). A systematic appraisal of the information, engagement, aesthetic and functional quality of nutrition-related smartphone apps for children and adolescents. Public Health Nutr.

[18] Stoyanov SR, Hides L, Kavanagh DJ, Zelenko O, Tjondronegoro D, Mani M (2015). Mobile App Rating Scale: a new tool for assessing the quality of health mobile apps. JMIR mHealth uHealth.

[19] Michie S, van Stralen MM, West R (2011). The behaviour change wheel: a new method for characterising and designing behaviour change interventions. Implement Sci.

[20] Eberhard DM, Simons GF, Robinson AJ (2026). English. Ethnologue: languages of the world.

[21] Parry G (2025). English language teaching in Africa (ELT): a comprehensive overview of ELT in Africa. Global Services in Education.

[22] Vailshery LS (2025). Google Play Store - statistics & facts. Statista.

[23] Slotta D (2025). Apple App Store - statistics & facts. Statista.

[24] Liberati A, Altman DG, Tetzlaff J (2009). The PRISMA statement for reporting systematic reviews and meta-analyses of studies that evaluate health care interventions: explanation and elaboration. PLOS Med.

[25] Anderson DK, Lord C, Risi S (2007). Patterns of growth in verbal abilities among children with autism spectrum disorder. J Consult Clin Psychol.

[26] Kim SH, Bal VH, Lord C (2018). Longitudinal follow-up of academic achievement in children with autism from age 2 to 18. J Child Psychol Psychiatry.

[27] Vogindroukas I, Stankova M, Chelas EN, Proedrou A (2022). Language and speech characteristics in autism. Neuropsychiatr Dis Treat.

[28] Suryawan A, Jalaludin MY, Poh BK (2022). Malnutrition in early life and its neurodevelopmental and cognitive consequences: a scoping review. Nutr Res Rev.

[29] Adams SN (2022). Feeding and swallowing issues in autism spectrum disorders. Neuropsychiatr Dis Treat.

[30] Adams SN, Verachia R, Coutts K (2022). Mealtime challenges and food selectivity in children with autism spectrum disorder in South Africa. S Afr J Clin Nutr.

[31] Adams SN, Matsimela N (2023). Feeding children with autism in South Africa: the teachers’ perspectives. Afr J Disabil.

[32] Kakooza-Mwesige A, Bakare M, Gaddour N, Juneja M (2022). The need to improve autism services in lower-resource settings. Lancet.

[33] Kuschner ES, Morton HE, Maddox BB, de Marchena A, Anthony LG, Reaven J (2017). The BUFFET program: development of a cognitive behavioral treatment for selective eating in youth with autism spectrum disorder. Clin Child Fam Psychol Rev.

[34] Sharp WG, Burrell TL, Jaquess DL (2014). The Autism MEAL Plan: a parent-training curriculum to manage eating aversions and low intake among children with autism. Autism.

[35] Clancy AM, O’Driscoll K, Regan J, Walsh IP (2025). Interventions for autistic children with behavioural feeding needs: a scoping review. Adv Autism.

[36] Martinon P, Saliasi I, Bourgeois D (2022). Nutrition-related mobile apps in the French app stores: assessment of functionality and quality. JMIR mHealth uHealth.

[37] Mummah SA, Robinson TN, King AC, Gardner CD, Sutton S (2016). IDEAS (Integrate, Design, Assess, and Share): a framework and toolkit of strategies for the development of more effective digital interventions to change health behavior. J Med Internet Res.

[38] Michie S, Yardley L, West R, Patrick K, Greaves F (2017). Developing and evaluating digital interventions to promote behavior change in health and health care: recommendations resulting from an international workshop. J Med Internet Res.

[39] Toda A, Klock ACT, Pereira FD, Rodrigues LA, Palomino PT, Lopes V Towards the understanding of cultural differences in between gamification preferences: a data-driven comparison between the US and Brazil. https://educationaldatamining.org/EDM2022/proceedings/2022.EDM-posters.60/index.html.

[40] Farinha J, de Fatima Pina M (2025). Evolving Strategies for Organizational Management and Performance Evaluation.

[41] Stoyanov SR, Hides L, Kavanagh DJ, Wilson H (2016). Development and validation of the user version of the Mobile Application Rating Scale (uMARS). JMIR mHealth uHealth.

