# Supplementary material for: Mobile Apps Addressing Feeding of Children With Autism: Systematic Search and Content and Quality Analysis
Source: JMIR Mhealth Uhealth. 2026 Nov 10;14:e94908. doi: 10.2196/94908 (PMC13750524; doi:10.2196/94908)
Supplement: Supplementary file 1 [file mhealth-v14-e94908-s001.docx]

# Multimedia Appendix 1. List of Screened Apps

Table S1: Screened Applications and Their Primary Focus (September–October 2024).

|  | **Apps Name** | **Platform** | **Category** | **Primary Focus** |
| --- | --- | --- | --- | --- |
| 1 | 2nd Grade Baby Book  Animal Flashcards For Kids or Kindergarten to Learn Words  With Sounds | iOS | Early Learning & Education | To help young children learn first  words and animal names through interactive flashcards with sounds |
| 2 | Aiko and Egor: Animation 4 Autism | Android | Autism Education & Skills Training | To promote learning through animated videos and games targeting key skills for individuals with autism |
| 3 | App4Autism-Timer,  Visual Planning | Android | Daily Living & Visual Scheduling | To help parents and teachers efficiently use tools like images and audios to work with children with  autism |
| 4 | ASDHelp: Kids’s  Autism Games | Android | Therapy & Skill Development | To help therapists track child’s  progress and suggest new exercises, and help parents reduce the effects of autism at home |
| 5 | ASDetect | Android | Autism Screening & Assessment | To help parents and caregivers review possible early signs of autism in children under 2.5 years |
| 6 | Autism 360 | Android | Parent & Caregiver Support | The parenting app for parenting  kids 1-18 in autism spectrum and associated conditions |
| 7 | Autism 911 | Android | Parent & Caregiver Support | To support families with autistic  children |
| 8 | Autism ABC App | Android | Autism Education & Skills Training | To provide a dedicated tool for  educators and parents of autistic children, offering both entertainment and therapeutic support |
| 9 | Autism AI | Android | Autism Screening & Assessment | An autistic traits detection system using AI |
| 10 | Autism Diary | Android | Behavior Tracking & Monitoring | To track daily habits and behaviors, helping caregivers manage the needs of individuals with  autism |
| 11 | Autism Evaluation  Checklist | Android | Autism Screening & Assessment | To help in testing for autistic  spectrum disorder |

| 12 | Autism Food Coach 2 | iOS | Feeding & Nutrition | To support parents in helping  children develop healthier eating habits through mindful eating practices |
| --- | --- | --- | --- | --- |
| 13 | Autism Parenting  Magazine | Android | Parent & Caregiver Support | To provide autism therapies, solutions, and news |
| 14 | Autism Social Video  Exercises | Android | Social Skills Training | Designed to build social confidence for individuals with ASD through interactive video exercises |
| 15 | Autism Speaks  Fundraising | Android | Fundraising & Advocacy | To help in fundraising by tracking progress and personalizing fundraising pages for autistic people |
| 16 | Autism Speech and  Language | Android | Speech & Language Therapy | For parents to help their autistic  children improve speech and language development |
| 17 | Autism Test (Adult) | Android | Autism Screening & Assessment | To evaluate the risk of autism  spectrum disorder with a validated screening test |
| 18 | Autism Test (Child) | Android | Autism Screening & Assessment | To test if a child is autistic |
| 19 | AutiSpark: Kids  Autism Games | Android | Autism Education & Skills Training | To provide fun learning activities  and games specially for kids with autism spectrum disorder |
| 20 | Autistapp | Android | General Autism Support | App to help autistic individuals |
| 21 | Autism: Daily Living  and Caring | Android | Daily Living & Caregiving | Designed for families and carers  to create a private group to help care for someone with autism |
| 22 | Autimo-AMIKEO  APPS | Android | Emotional Recognition & Social Skills | To help children with autism understand emotions with enjoyable activities |
| 23 | Awetism Insights | Android | Parent & Caregiver Support | App designed to support parents  of autistic children |
| 24 | Car Puzzle 2 for toddlers | iOS | Early Learning & Education | To help toddlers, including those  with autism, learn about cars and transportation through interactive puzzles |
| 25 | Easy jigsaw puzzle  games for toddlers  and babies | iOS | Early Learning & Education | Made for kids aged 1-4 to introduce food and kitchen items  through puzzles and mini-games |

| 26 | EduKitchen-Toddlers  Food Games | iOS | Feeding & Nutrition | To teach children aged 2-6 healthy eating, counting, and logic through kitchen-themed games |
| --- | --- | --- | --- | --- |
| 27 | Flashcards for kids-  First Food Words | iOS | Early Learning & Education | To provide customizable, interactive flashcards for early childhood learning (ages 0-6) |
| 28 | Fruits and Vegetables  Flashcards quiz and matching game for toddlers and kids in  English | iOS | Early Learning & Education | To help kids learn names of fruits and vegetables through interactive games |
| 29 | Fun Routine-Autism | Android | Daily Living & Routines | To manage daily routines, Improve communication, and strengthen socio-emotional development |
| 30 | iDo Food-Kids with  Special needs learn dining skills (Full Version) | iOS | Feeding & Nutrition | To teach cooking/dining skills for individuals with autism and intellectual disabilities |
| 31 | Jade | Android | Therapy & Skill Development | For children/adolescents with autism, developmental delay, or learning difficulties |
| 32 | Language Therapy for  Children | Android | Speech & Language Therapy | To provide early intervention language therapy for children with delays and autism |
| 33 | Leeloo AAC-Autism  Speech App | Android | Augmentative & Alternative Communication (AAC) | To help non-verbal children communicate using visual cards (AAC/PECS) |
| 34 | Learn Autism | Android | Autism Education & Resources | Free, on-demand videos created by Autism experts |
| 35 | Matraquinha: Autism | Android | Augmentative & Alternative Communication (AAC) | To help autistic people with communication |
| 36 | Meet the Colors | iOS | Early Learning & Education | To help toddlers learn colors through interactive games |
| 37 | Meet the Letters Lowercase | iOS | Early Learning & Education | To help toddlers learn lowercase letters through matching games |
| 38 | Meet the Letter Uppercase | iOS | Early Learning & Education | To help toddlers learn uppercase letters through interactive play |
| 39 | Meet the Numbers | iOS | Early Learning & Education | To help toddlers learn numbers 0-10 through tapping games |

| 40 | Meet the Shapes | iOS | Early Learning & Education | To teach toddlers about shapes using interactive games |
| --- | --- | --- | --- | --- |
| 41 | Music Therapy for  Autism | Android | Therapy & Intervention | To help children with ASD and ADHD |
| 42 | My Autism Navigator | Android | Parent & Caregiver Support | To support autistic children’s learning in everyday activities |
| 43 | NeuroSchemas for Autism | Android | Social Skills Training | To help individuals learn social rules for different situations |
| 44 | Otsimo | Android | Special Education | Assistive technology for special education |
| 45 | Otsimo-Special Education | Android | Special Education | Designed for individuals with autism, Down syndrome, and other special needs |
| 46 | Otsimo-Speech Therapy SLP | Android | Speech & Language Therapy | To help kids with speech issues, including autism |
| 47 | Otsimo AAC-Tap and  Talk | Android | Augmentative & Alternative Communication (AAC) | AAC speech solution for individuals with communication difficulties |
| 48 | Pediatric Nutrition | Android | Nutrition Education | Evidence-based guidance on childhood nutrition issues |
| 49 | POOW The Food  Hero | iOS | Feeding & Nutrition | To assist parents of picky eaters (ages 2-5) to try new flavors |
| 50 | Pravah: Autism Care  Activities | Android | Therapy & Intervention | Personalized therapy activities for Autism/ADHD |
| 51 | SenLife-ADHD and  Autism Support | Android | Behavior Tracking & Monitoring | To help parents track behavior of children with ADHD or ASD and create reports for home or school care |
| 52 | Sight Words 1 Flashcards | iOS | Early Learning & Education | To teach toddlers sight words through interactive flashcards |
| 53 | Sight Words 1 Guessing Game | iOS | Early Learning & Education | To help toddlers learn 16 sight words through flashcards |
| 54 | Sight Words 2 Flashcards | iOS | Early Learning & Education | To teach toddlers sight words using interactive flashcards |
| 55 | Sight Words 3 Flashcards Sight Words 2 Guessing Game | iOS | Early Learning & Education | To help toddlers learn sight words through interactive games |
| 56 | Sight Words 3 Guessing Game | iOS | Early Learning & Education | To teach sight words with tappable characters and animations |

| 57 | TalkTablet PRO  Autism and Stroke | Android | Augmentative & Alternative Communication (AAC) | Comprehensive AAC speech solution for communication difficulties |
| --- | --- | --- | --- | --- |
| 58 | The Autism Helper | Android | Parent & Caregiver Support | To support educators and parents of autistic individuals |
| 59 | ThinkAutism | Android | Autism Education & Screening | To learn about Asperger’s and complete a screening questionnaire |
| 60 | Tiny Tastes | iOS | Feeding & Nutrition | To encourage picky eaters to try new foods through games |
| 61 | Verbal Autism | Android | Augmentative & Alternative Communication (AAC) | Designed for nonverbal children on the Autism Spectrum |
| **Newly Identified Applications and Their Primary Focus from the June-July 2026 Screening (Not Identified in the September-October 2024 Screening)** | | | | |
|  | **Apps Name** | **Platform** | **Category** | **Primary Focus** |
| 1 | TummyTurtle | iOS | Feeding & Nutrition | To encourage positive eating behaviors and food exploration in young children through interactive activities and gamified food exposure. |
| 2 | Food Hopper | iOS | Feeding & Nutrition | To promote children's willingness to try new foods and support food exploration through interactive games and rewards. |
| 3 | Little Spoon | iOS | Child Nutrition & Meal Planning | To provide healthy meal planning and nutrition guidance for infants, toddlers, and young children. |
| 4 | Spoonful | iOS | Food Allergy & Nutrition | To help families identify allergens and make informed food choices by scanning food products. |
| 5 | FoodIQ | iOS | Nutrition Education | To improve children's nutrition knowledge and healthy eating habits through educational activities. |
| 6 | ARFID & Picky Eaters | iOS | Feeding Disorders | To support individuals with avoidant/restrictive food intake disorder (ARFID) and picky eating by providing structured feeding guidance and educational resources. |
| 7 | 101 before one | iOS | Infant Feeding & Baby-Led Weaning | To guide parents on introducing complementary foods before one year of age using evidence-based recommendations. |
| 8 | Choicelunch | iOS | School Meal Management | To allow parents to order and manage healthy school meals for children. |
| 9 | Feeding Littles: Meal Planner | iOS | Feeding & Nutrition | To assist parents in planning balanced meals and provide evidence-based feeding guidance for infants and young children. |
| 10 | Little Lunches: Kids Meals | iOS | Meal Planning | To help parents prepare healthy lunchboxes and balanced meals for children. |
| 11 | Nutrislice | iOS | School Nutrition | To provide school menus, nutrition information, and meal planning resources. |
| 12 | Kids Cooking Toddler Games | iOS | Feeding & Nutrition | To teach toddlers about food preparation, cooking, and healthy eating through interactive educational games. |
| 13 | Starting Solids: Baby Food | iOS | Infant Feeding | To guide parents through introducing solid foods and complementary feeding safely. |
| 14 | My Little Food Critic | iOS | Feeding & Nutrition | To provide practical advice and recipes for feeding babies and toddlers. |
| 15 | BLW Meals: Starting Solids | iOS | Baby-Led Weaning | To support parents practicing baby-led weaning with meal ideas and feeding guidance. |
| 16 | Baba – Toddler Meals | iOS | Toddler Nutrition | To provide healthy meal plans and recipes for toddlers. |
| 17 | Annabel Karmel Kids Recipes | iOS | Child Nutrition & Recipes | To offer healthy recipes, meal ideas, and nutrition guidance for babies and children. |
| 18 | ReciMe: Recipes & Meal Planner | iOS | Meal Planning | To organize recipes and create personalized meal plans. |
| 19 | Fooducate: Nutrition Coach | iOS | Nutrition Education | To help users make healthier food choices through nutrition tracking and food ratings. |
| 20 | BetterBites – Kids Eat in Color | iOS | Feeding & Nutrition | To provide caregivers with nutrition education, meal-planning resources, and practical strategies for encouraging healthy eating habits in children. |
| 21 | Solid Starts: Baby First Foods | iOS | Infant Feeding & Baby-Led Weaning | To guide parents on introducing first foods safely and reducing picky eating. |
| 22 | Smoosh: Toddler Meal Planner | iOS | Toddler Nutrition | To generate balanced meal plans and recipes for toddlers. |
| 23 | All Done: Toddler Nutrition | iOS | Toddler Nutrition | To support healthy nutrition and meal tracking for toddlers. |
| 24 | MealManage | iOS | Meal Planning & Nutrition | To assist families with meal planning, grocery organization, and nutrition management. |
